# An endogenous promoter *LpSUT2* discovered in duckweed: a promising transgenic tool for plants

**DOI:** 10.3389/fpls.2024.1368284

**Published:** 2024-04-03

**Authors:** Cuicui Wei, Zhubin Hu, Songhu Wang, Xiao Tan, Yanling Jin, Zhuolin Yi, Kaize He, Leyi Zhao, Ziyue Chu, Yang Fang, Shuang Chen, Penghui Liu, Hai Zhao

**Affiliations:** ^1^ CAS Key Laboratory of Environmental and Applied Microbiology, Environmental Microbiology Key Laboratory of Sichuan Province, Chengdu Institute of Biology, Chinese Academy of Sciences, Chengdu, China; ^2^ University of Chinese Academy of Sciences, Beijing, China; ^3^ Anhui Province Key Laboratory of Horticultural Crop Quality Biology, School of Horticulture, Anhui Agricultural University, Hefei, China; ^4^ Pitzer College, Claremont, CA, United States; ^5^ Faculty of Mathematical and Physical Sciences, University College London, London, United Kingdom; ^6^ School of Breeding and Multiplication (Sanya Institute of Breeding and Multiplication), Hainan University, Sanya, China

**Keywords:** endogenous strong promoter, 35S promoter, LpSUT2 promoter, duckweed, antibiotic stress

## Abstract

Promoters are one of the most critical elements in regulating gene expression. They are considered essential biotechnological tools for heterologous protein production. The one most widely used in plants is the *35S* promoter from *cauliflower mosaic virus*. However, our study for the first time discovered the *35S* promoter reduced the expression of exogenous proteins under increased antibiotic stress. We discovered an endogenous strong promoter from duckweed named *LpSUT2* that keeps higher initiation activity under antibiotic stress. Stable transformation in duckweed showed that the gene expression of *eGFP* in the *LpSUT2:eGFP* was 1.76 times that of the *35S:eGFP* at 100 mg.L^-1^ G418 and 6.18 times at 500 mg.L^-1^ G418. Notably, with the increase of G418 concentration, the gene expression and the fluorescence signal of *eGFP* in the *35S:eGFP* were weakened, while the *LpSUT2:eGFP* only changed slightly. This is because, under high antibiotic stress, the *35S* promoter was methylated, leading to the gene silencing of the *eGFP* gene. Meanwhile, the *LpSUT2* promoter was not methylated and maintained high activity. This is a previously unknown mechanism that provides us with new insights into screening more stable promoters that are less affected by environmental stress. These outcomes suggest that the *LpSUT2* promoter has a high capacity to initiate the expression of exogenous proteins. In conclusion, our study provides a promoter tool with potential application for plant genetic engineering and also provides new insights into screening promoters.

## 1 Introduction

Promoters are one of the most critical regulatory elements controlling gene expression in terms of time and space. It is an invaluable and indispensable tool for investigating specific gene functions, crop yield improvement, plant stress resistance, heterologous protein production, etc (Nuccio, 2018). Promoters are widely used in synthetic biology and plant biotechnology. Many plants have been widely used as expression systems for the commercial production of heterologous proteins for their lower production cost and easier to scale-up (Sharma and Sharma, 2009; Tiwari et al., 2009).

So far, the earliest and most frequently used promoter in plant bioreactors is the *35S* promoter from *cauliflower mosaic virus* (Nuccio, 2018; Tian et al., 2022). Examples of such applications include the envelope protein of Japanese encephalitis virus expressed in rice (Wang et al., 2009), *CTB-InsB3* expressed in tobacco (Tiwari et al., 2009), human β-amyloid expressed in potato (Kim et al., 2003), etc. In addition, the *35S* promoter was widely used to drive the over-expression of key genes to improve crop yield. For example, the *35S* promoter driven over-expression of plasma membrane genes, such as *H^+^ATPase*, *MFAP1*, *NAC23*, *FTO*, and H^+^-pyrophosphatase *IbVP1*, increased rice, potato, and sweet potato yield, respectively (Fan et al., 2021; Yu et al., 2021; Zhang et al., 2021; Y. Li et al., 2022; Z. Li et al., 2022). The *35S* promoter also plays an important role in the over-expression of the plant stress resistance genes. Subsequently, *VvCEB1opt* over-expression increases salinity tolerance in *Arabidopsis* (Lim et al., 2020) and *IbPSS1* over-expression enhances salt tolerance in transgenic sweet potatoes at the whole-plant level (Yu et al., 2020). Nevertheless, limitations of the *35S* promoter have been discovered. These include disturbing the expression of adjacent genes and their low activity in monocots (Peremarti et al., 2010; Nuccio, 2018; Somssich, 2019). Thus, researchers tended to focus on finding endogenous strong promoters in plants and discovered rice actin 1 (*Actin1*) and maize ubiquitin1 (*Ubi1*) promoters (Park et al., 2010; Nuccio, 2018). However, these promoters are only widely used in monocots (Wu et al., 2019), and their activity level decreases with the development of plants (Nuccio, 2018). Furthermore, experiments using the *35S* promoter and maize *Ubi1* promoter suggest that is difficult to improve their activity (Nuccio, 2018). Therefore, it is necessary and urgent to find a new endogenous strong promoter to express heterologous proteins, increase plant stress resistance, and improve crop yield, that applies to both monocots and dicots plants.

Duckweed, the smallest monocotyledonous group of flowering aquatic plants spreads worldwide, comprises 5 genera (*Spirodela*, *Landoltia*, *Lemna*, *Wolffia*, and *Wolffiella*) and 36 species (Bog et al., 2019). It grows fast, does not compete for land with crops, and has a high protein content (Liu et al., 2015; Tian et al., 2021). Thus, duckweed has been widely used as a bioreactor or biosynthetic chassis to produce recombinant proteins (Lam and Michael, 2022). Previous studies used duckweed as an expression system to produce protective antigen (Ko et al., 2011), M130-β-glucuronidase (Firsov et al., 2015), hemagglutinin (Bertran et al., 2015), recombinant human granulocyte colony-stimulating factor (Khvatkov et al., 2018), etc. Nevertheless, all the above studies expressed heterologous proteins with the *35S* promoter. Using endogenous promoters for expressing recombinant proteins reduces potential risks associated with exogenous promoters and has demonstrated favorable outcomes. For instance, the utilization of the rice endosperm-specific *Gt13a* promoter drove the expression of human serum albumin and recombinant human basic fibroblast growth factor in rice seeds, showcasing the capacity of this system for large-scale production of functional human recombinant proteins (He et al., 2011; An et al., 2013). However, limited research has been dedicated to utilizing the endogenous strong promoter of duckweed for expressing heterologous or recombinant proteins. For these reasons, we aimed to screen endogenous strong promoters of duckweed for application in transgenic technology to augment the expression of exogenous proteins and explore potential molecular mechanisms. Duckweed exhibits a simple leaf–stem structure known as a frond, yet it demonstrates rapid growth, high starch accumulation capacity, and exceptional photosynthetic efficiency (Chen et al., 2022; Guo et al., 2023). In various plant species, promoters such as the sucrose transporter 2 promoter, Rubisco promoter, ubiquitin promoter, and others have demonstrated favorable outcomes in transgenic technology (Christensen and Quail, 1996; Stadler and Sauer, 2019; Boruah et al., 2023). Taking these factors into account, we considered the ADP-glucose pyrophosphorylase small subunit 1 (*AGPS1*) promoter, sucrose transporter 2 promoter, Rubisco promoter, and ubiquitin promoter in duckweed as candidate promoters for screening endogenous strong promoters in duckweed.

In this work, we found the duckweed sucrose transporter 2 promoter of *L. punctata* (*LpSUT2*) is an endogenous strong promoter, and expresses the exogenous proteins higher than the *35S* promoter in duckweed, a monocot. It also has the same high activity as the *35S* promoter in *Nicotiana tabacum*, a dicot. Increasing antibiotic stress can reduce the escape in transgenic events (Shin et al., 2007; Itaya et al., 2018). We discovered the *LpSUT2* promoter behaved very steadily even under high antibiotic stress, while the *35S* promoter did not. Together, our research offers a promoter tool with stable and effective function and application value for plant biotechnology.

## 2 Materials and methods

### 2.1 Plant materials

*Landoltia punctata* 0202 line and *Lemna minor* ZH0403 callus were stored in the duckweed resource bank at the Chengdu Institute of Biology, Chinese Academy of Science (Li et al., 2021; Tian et al., 2021). Genomic DNA was extracted from the *Landoltia punctata* 0202 line cultured in Hoagland medium (15 g.L^-1^ sucrose, pH 5.00 ± 0.05). The callus was cultured in Murashige and Skoog (MS) medium with 0.22 mg.L^-1^ 2,4-Dichlorophenoxyacetic acid, 0.45 mg.L^-1^ 6-Benzylaminopurine, 30 g.L^-1^ sucrose, and 3.5 g.L^-1^ gellan gum and then used for genetic transformation. These mediums were placed in a system of photon flux density cycle of 100-120/0 µmol^-2^s^-1^ (16 h light/8 h dark) and a temperature cycle of 25°C/15°C (16 h day/8 h night).

### 2.2 Construction of vectors

The 2000 bp promoter DNA sequences upstream from specific genes, sucrose transporter 2 gene promoter named *LpSUT2* (Landoltia_punctata_GLEAN_10005267), *AGPS1* gene promoter named *LpA6804* (Landoltia_punctata_GLEAN_10016804), Rubisco1 gene promoter named *LpR1090* (Landoltia_punctata_GLEAN_10021090), Rubisco2 gene promoter named *LpR1091* (Landoltia_punctata_GLEAN_10021091), ubiquitin-like protein gene promoter named *LpU4817* (Landoltia_punctata_GLEAN_10004817), and ubiquitin-1 promoter named *LpU9400* (Landoltia_punctata_GLEAN_10019400) were extracted from the genome of *L. punctata* by TBtools software (Chen et al., 2020). The cetyltrimethylammonium bromide (CTAB) method was used to extract DNA from *L. punctata* (Allen et al., 2006). Next, DNA sequences of these promoters were cloned using high PCR fidelity KOD Plus enzymes (TOYOBO, Japan) and recombinant primers (**Supplementary Table 1**). These amplified products and the linearized vector (the binary vector pCambia2301:*35S:eGFP* were digested by the restriction enzymes *Pvu*II and *Pst*I) were connected by ClonExpress^®^ MultiS One Step Cloning Kit (Vazyme, Nanjing, China) and then sequenced.

### 2.3 Western blot analysis

We referenced previous studies for the protein extraction methods of transgenic duckweed and non-transgenic duckweed (Khvatkov et al., 2018). According to the previously established method (Mahmood and Yang, 2012; Khvatkov et al., 2018), we detected the 35S: ChIL-2:His protein by the Western blot at 100 mg.L^-1^ and 500 mg.L^-1^ geneticin (G418) from the *35S: ChIL-2:His* transgenic duckweed (data were not published). Anti-HIS mouse monoclonal antibody (diluted 1:2,000; Sangon Biotech, China) and Anti-HRP rabbit polyclonal antibody (diluted 1:10,000; Sangon Biotech, China) were used in this work. The blots were visualized using ECL Western blotting detection reagents (Cytiva, America).

### 2.4 Transient expression in dicot *Nicotiana tabacum* leaves

Promoter activity was often studied directly using a reporter gene (*eGFP* or *GUS*) in stable or transient transformation assays (Nuccio, 2018). As a reporter gene, β-glucuronidase (*GUS*) is suitable for macroscopic observation of tissue structure in promoter-initiated gene expression. However, it is not suitable for real-time observation of gene expression due to its requirement for steps such as hydrolysis reaction and destaining. On the other hand, enhanced green fluorescent protein (*eGFP*), as a reporter gene, enables precise mapping at the living cell level, making it suitable for observing the more subtle real-time positional structure of promoter-initiated gene expression. Therefore, we chose *eGFP* as the reporter gene to provide a more detailed insight into the gene expression initiated by the candidate promoters. To investigate the candidate promoters activity in dicot, we used *35S*, *LpSUT2*, *LpR1091*, *LpA6804*, *LpU9400*, *LpR1090*, and *LpU4817* promoters to respectively drive the reporter gene *eGFP*, and transiently expressed each in *Nicotiana tabacum* leaves and left for 2 days. This method was carried out as described in previous studies (Yao et al., 2015). Then we used a laser scanning confocal microscope to observe the fluorescence signal of eGFP.

### 2.5 Stable expression in the monocot duckweed

The vectors of the *35S:eGFP* and the *LpSUT2:eGFP* were introduced into duckweed’s callus following *Agrobacterium tumefaciens* strain GV3101-mediated transformation. Then, follow the previously established method to screen and regenerate these calluses (Tan et al., 2022). Briefly, put these callus in MS medium (screening 6 weeks) and 1/2 Schenk and Hildebrandt (SH) medium (regeneration 4-5 weeks). Specially, the two-stage culture medium (MS for screening and 1/2 SH for regeneration) contained G418 concentrations of 50, 100, 200, 300, 400, and 500 mg.L^-1^, respectively.

### 2.6 Isolation of RNA and qRT−PCR analysis

Total RNAs were extracted using the Eastep Super Total RNA Extraction Kit (Promega, Shanghai, China) according to the manufacturer’s instructions. Next, we examined their concentration and quality by NanoDrop 2000 (Thermo, USA). All RNA samples were treated with RNase-free DNase to eliminate DNA contamination. The cDNA was synthesized from 1 μg of total RNAs following the manufacturer’s protocol of the GoScript™ Reverse Transcription System (Promega, Shanghai, China). These samples were harvested and immediately frozen in liquid nitrogen, subsequently stored in a -80°C freezer until being used. The *L. minor* 18S gene was used as an internal control. Primer sequences were listed in **Supplementary Table 1**. The qRT-PCR was operated by CFX Maestro Real-Time PCR (Bio-Rad, USA), using the SsoAdvanced™ Universal SYBR Green Supermix (Bio-Rad, USA). The PCR reaction system, procedures, and data analysis followed the SsoAdvanced™ Universal SYBR Green Supermix protocol.

### 2.7 Methylation analysis

We treated the *35S:eGFP* transgenic duckweed at 500 mg.L^-1^ G418 with 45.64 mg.L^-1^ zebularine (a methylation inhibitor) for 8 days. Then, the transgenic duckweed from the other treatment groups was collected together. Transgenic duckweed genomic DNA was extracted using the CTAB method. The methylation status of promoters was determined by methylation-specific PCR. Briefly, 2 μg of genomic DNA was bisulfite-treated with the Zymo DNA Modification Kit (Zymo Research, Orange County, CA, USA). Bisulfite-treated DNA was amplified using primers specific for either methylated or unmethylated DNA (Baik et al., 2021). The sequences of the first set of methylated-specific (M) primers and unmethylated-specific (U) primers for the promoters are listed in **Supplementary Table 1**.

### 2.8 Fluorescence signal detection

In this work, the fluorescence signal of eGFP protein in all transgenic plants was observed using a laser scanning confocal microscope. To maintain the consistency of the parameters in the obtained fluorescence images, all parameters were set as follows: equipment, Leica TCS SP8; excitation and emission wavelengths, Ex488 nm/Em505-530 nm; software, LAS AF; values of intensity, 30%; the master gain, 800 V; digital gain, 1; pinhole, 1 AU.

### 2.9 Data analysis

We used GraphPad Prism (Version 8.0) and SPSS 26 software for data analysis.

## 3 Results

### 3.1 Screening of the endogenous strong promoter of duckweed

**Figure 1:**
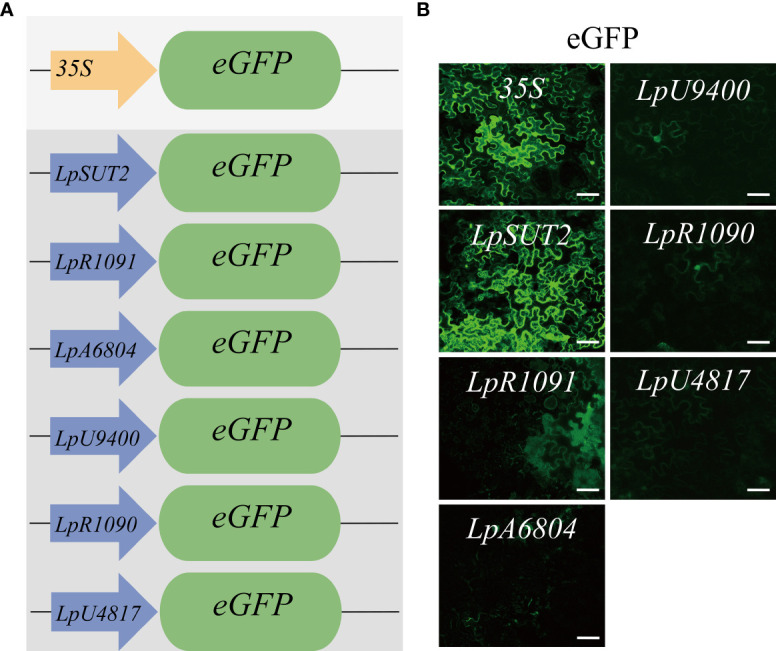
Initiating activity of the promoters in dicotyledonous *Nicotiana tabacum* leaves. **(A)** Schematic representation of the vector structures. The expression of the *eGFP* gene was driven by the *35S* promoter and duckweed endogenous promoters (*LpSUT2*, *LpR1091*, *LpA6804*, *LpU9400*, *LpR1090*, and *LpU4817* promoter). **(B)** The binary vectors pCambia2301:the *35S*:*eGFP*, pCambia2301:*LpSUT2*:*eGFP*, pCambia2301: *LpR1091*:*eGFP*, pCambia2301: *LpA6804*:*eGFP*, pCambia2301: *LpU9400*:*eGFP*, pCambia2301: *LpR1090*:*eGFP*, and pCambia2301: *LpU4817*:*eGFP* were transiently expressed in *Nicotiana tabacum* leaves for 2 days. The green signals indicate eGFP. Scale bars: 50 μm.

To find a promoter with wide application potential in plants, we selected six duckweed endogenous promoters in contrast to the widely utilized *35S* promoter from *cauliflower mosaic virus*. In parallel, we used the *35S* promoter and replaced the *35S* promoter with the candidate promoters to drive the *eGFP* gene into the binary vector pCambia2301 (**Figure 1A**). To test whether the candidate promoters from monocots were suitable in dicots, we transiently expressed these plasmids were transiently expressed in dicots *Nicotiana tabacum* leaves for 2 days with *Agrobacterium tumefaciens*-mediated infiltration (Batistič et al., 2012). The images were acquired using consistent acquisition parameters. Under the same validation conditions, we observed that only the eGFP fluorescence signal initiated by the *LpSUT2* promoter exhibited comparable intensity to that of the *35S* promoter, indicating similar strong initiation abilities. The weaker eGFP fluorescence signals were initiated by the other endogenous promoters (*LpR1091*, *LpA6804*, *LpU9400*, *LpR1090*, and *LpU4817* promoter) (**Figure 1B**). It is possible that they have a limited initiation ability or tissue-specific expression; further studies are necessary to distinguish these possibilities. Based on this result, only the *LpSUT2* promoter fulfilled our objective of screening endogenous promoters with strong initiation ability and practicality. Subsequently, we concentrated on comparing the initiation activity and stability of the *LpSUT2* promoter and the *35S* promoter in duckweed.

### 3.2 Stable expression in duckweed, a monocot

**Figure 2:**
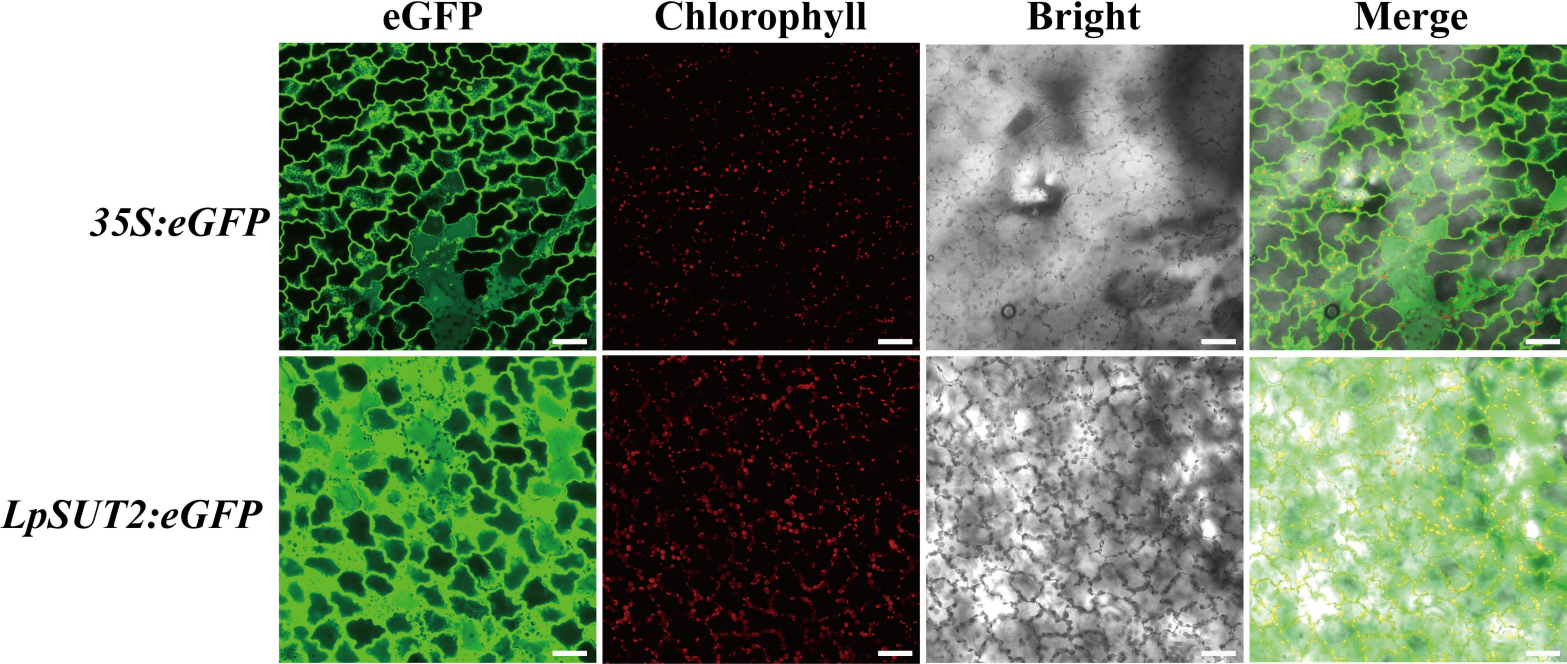
Comparison of the *LpSUT2* and the *35S* promoter activities in monocotyledonous duckweed fronds. The *35S*:*eGFP* and the *LpSUT2*:*eGFP* in transgenic leaves of duckweed. The green signals indicate eGFP and the red signals indicate chlorophyll. Scale bars: 50 μm.

Recent studies showed that duckweed has been a widely used bioreactor to express the alpha-interferon, M2e peptide of avian influenza virus H5N1 (Cox et al., 2006; Firsov et al., 2015), etc. Meanwhile, the *35S* promoter has been proven to drive the expression of the target proteins in duckweed. However, the *35S* promoter also raises concerns about its biosafety since it comes from a virus (Ho et al., 1999), and the low activity in the cell to recognize the sequence as foreign inactivates it (Elmayan and Vaucheret, 1996). Therefore, we expect that finding a plant endogenous promoter will become a more effective tool to produce recombinant proteins. To evaluate the activity of the *LpSUT2* promoter in monocot duckweed, we stably transformed the *35S:eGFP* (positive control) and the *LpSUT2:eGFP* vectors into duckweed by *Agrobacterium tumefaciens* mediated infiltration. Geneticin G418 is an aminoglycoside antibiotic that inhibits protein synthesis by binding to the 30S subunit of the ribosome of a cell. G418 is commonly employed in transgenic plant screening. In our stable transformations in duckweed, G418 was also employed as an antibiotic selection marker. We followed the G418 resistance marker carried by the pCambia2301. Consequently, plants were selected by the addition of G418 to the medium. We collected the images under the same acquisition parameters. Notably, the *LpSUT2* promoter was highly active in transgenic duckweed fronds and stronger than the *35S* promoter by 100 mg.L^-1^ G418 screening (**Figure 2**).

### 3.3 The *LpSUT2* promoter drives high-level *eGFP* expression

**Figure 3:**
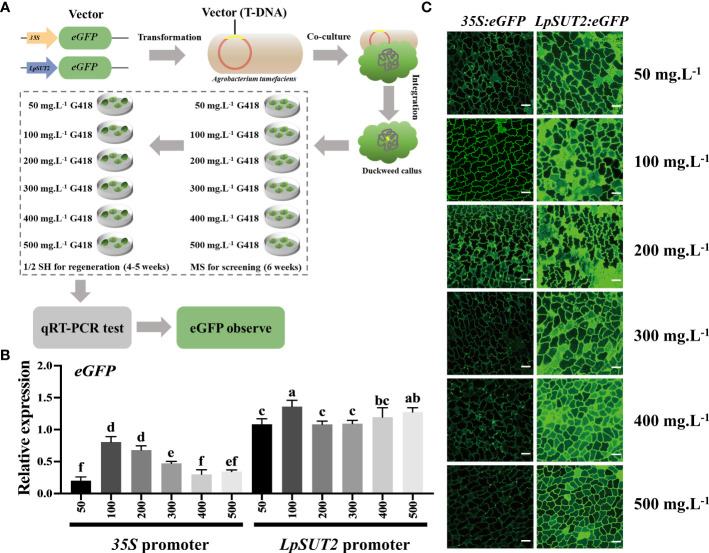
The ability of the *LpSUT2* and the *35S* promoters to initiate *eGFP* gene expression under different antibiotic stresses. **(A)** The recombinant plasmids the *LpSUT2*:*eGFP* and *35S*:*eGFP* were transformed into duckweed callus. Screened (MS medium) and regenerated (1/2 SH medium) the transgenic duckweed in these culture medium at 50, 100, 200, 300, 400, and 500 mg.L^-1^ G418, respectively. The gene expression level and the fluorescence intensity of *eGFP* were detected by qRT-PCR and laser scanning confocal microscope, respectively. The *eGFP* expression levels **(B)** and the fluorescence signals of the eGFP **(C)** in the *35S*:*eGFP* and the *LpSUT2*:*eGFP* transgenic lines grown in medium containing 50, 100, 200, 300, 400, and 500 mg.L^-1^ G418 were detected by qRT-PCR and laser scanning confocal microscope. Scale bars: 50 μm. For B, the 18S gene was used as an internal control. Data are the means ± SD of *n*=3. The letters indicate a significant difference between groups (*P* < 0.05 by one-way ANOVA followed by Tukey’s test).

To improve the efficiency of screening transgenic positive plants and test the stability of the promoter after increasing antibiotic stress, we obtained multiple stable transgenic lines using six concentrations of G418 (50, 100, 200, 300, 400, and 500 mg.L^-1^, respectively) during the screening and regeneration process (**Figure 3A**). Next, we used quantitative real-time PCR (qRT-PCR) on the stable transgenic lines from each of the six concentrations to detect the ability of promoters to initiate *eGFP* expression. The results showed that the expression level of *eGFP* driven by the *35S* promoter decreased with increasing G418 concentration, while the expression level of *eGFP* driven by the *LpSUT2* promoter did not significantly change and was all higher than that driven by the *35S* promoter under each condition. Indeed, the expression level of *eGFP* in the *LpSUT2*:*eGFP* transgenic duckweed was 1.76 times that of the *35S*:*eGFP* transgenic duckweed at 100 mg.L^-1^ G418, and 6.18 times at 500 mg.L^-1^ G418 (**Figure 3B**). Then, we observed the fluorescence signal of the *eGFP* protein using the laser scanning confocal microscope. The images were collected under the same parameters. Similar to the above qRT-PCR results, the fluorescence signal of the *LpSUT2*:*eGFP* transgenic lines had no significant change. However, the fluorescence intensity of the *35S*:*eGFP* transgenic lines became weaker as the concentration of G418 increased. More importantly, the *LpSUT2*:*eGFP* transgenic lines fluorescence intensity was consistently stronger than that of the *35S*:*eGFP* transgenic lines in each G418 concentration (**Figure 3C**). Therefore, these data demonstrate that the activity of the *LpSUT2* promoter is higher than that of the *35S* promoter in the monocotyledonous duckweed.

Based on the above interesting phenomenon, we hypothesized that the ability of the *35S* promoter to initiate the expression of foreign proteins would decrease after increasing the antibiotic stress. To test the hypothesis, we used seven transgenic duckweeds with the *35S* promoter driving chicken interleukin 2 (ChIL-2): His fusion protein in the pCambia2301 in the laboratory and cultured them at 100 or 500 mg.L^-1^ concentrations of G418 (with none other treatments). Interestingly, similar to the *35S*:*eGFP* results, we found that none of the ChIL-2:His fusion proteins in the transgenic duckweed were detectable when the G418 concentration changed from 100 mg.L^-1^ to 500 mg.L^-1^ (**Supplementary Figure 1**). Hence, these results indicate that the *35S* promoter initiation capacity is unstable under antibiotic stress, which is not conducive to increasing antibiotic stress to rapidly screen positive transgenic plants. In contrast to the *35S* promoter, the *LpSUT2* promoter exhibits a very stable initiation ability (**Figure 3C**).

### 3.4 Methylation analysis in the *35S* promoter and the *LpSUT2* promoter

Based on the previous experimental results, the expression of the *35S:eGFP* decreased with increasing concentration of G418, while the *LpSUT2:eGFP* remained stable. However, all transgenic duckweed lines exhibit phenotypic similarity to the wild type. There was also not much difference between 100 and 500 mg.L^-1^ G418 concentrations in the *35S:eGFP* or *LpSUT2:eGFP* transgenic duckweed phenotypes. Duckweed did not exhibit abnormal growth phenotypes, such as regional albino or death (**Supplementary Figure 2**).

**Figure 4:**
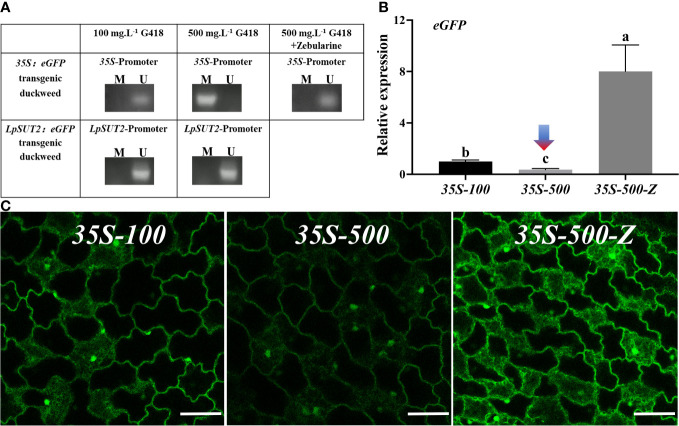
Methylation analysis of promoters. **(A)** Detection of DNA methylation in *35S* and *LpSUT2* promoter regions by methylation-specific PCR. M/U represents the amplified by methylation-specific primers or unmethylated-specific primers. The expression of the *eGFP* gene **(B)** and the fluorescence signal of the eGFP **(C)** in the *35S*:*eGFP* transgenic duckweed under different conditions. Scale bars: 50 μm. For **(B)**, the 18S gene was used as an internal control. Data are the means± SD of *n*=3. The letters indicate a significant difference between groups (*P* < 0.05 by one-way ANOVA followed by Tukey’s test). For **(B, C)**, *35S-100* represents *35S*:*eGFP* transgenic duckweed grown in 100 mg.L^-1^ G418. *35S-500* represents *35S*:*eGFP* transgenic duckweed grown in 500 mg.L^-1^ G418. *35S-500-Z* represents *35S*:*eGFP* transgenic duckweed grown in 500 mg.L^-1^ G418 with 45.64 mg.L^-1^ zebularine.

In *Arabidopsis*, the *35S* promoter was methylated, leading to the suppression of the overexpressed exogenous proteins (Lang et al., 2015). We predicted that the *35S* promoter methylation would cause the down-regulation of *eGFP* expression. To test this prediction, we utilized methylation-specific PCR to detect methylation in the *35S* promoter region in *35S:eGFP* transgenic duckweed lines grown in 100 mg.L^-1^ and 500 mg.L^-1^ G418, as well as in the *LpSUT2* promoter region in *LpSUT2:eGFP* transgenic duckweed lines grown in 100 mg.L^-1^ and 500 mg.L^-1^ G418. The results showed that methylation of the *35S* promoter region in the *35S:eGFP* transgenic duckweed line grown in 500 mg.L^-1^ G418, and no methylation was detected in other transgenic duckweed lines (**Figure 4A**).

Zebularine is a potent DNA methyltransferase inhibitor known to reduce DNA methylation in plants (Baubec et al., 2009). In our study, we supplemented 45.64 mg.L^-1^ Zebularine to inhibit methylation in the *35S:eGFP* transgenic duckweed line continuously grown in 500 mg.L^-1^ G418. After adding the methylation inhibitor, we found a reversion of the *35S* promoter region to a non-methylated state (**Figure 4A**).

Additionally, to assess the impact of DNA methylation on the initiation ability of the *35S* promoter, we quantified the expression levels of *eGFP* in the following groups: *35S-100*, representing *35S*:*eGFP* transgenic duckweed line grown in 100 mg.L^-1^ G418; *35S-500*, representing *35S*:*eGFP* transgenic duckweed line grown in 500 mg.L^-1^ G418; *35S-500-Z*, representing *35S*:*eGFP* transgenic duckweed line grown in 500 mg.L^-1^ G418 with 45.64 mg.L^-1^ zebularine. Compared with the *35S-100*, the expression of the *eGFP* gene was down-regulated by 2.73 times in the *35S-500* and up-regulated by 8.01 times in the *35S-500-Z* (**Figure 4B**). Then, we also observed the eGFP fluorescence signal in these groups. All images were acquired under the same parameters. Consistent with the above qRT-PCR results, *35S-500* shows a weaker fluorescence signal, and *35S-500-Z* shows a stronger fluorescence signal compared to *35S-100* (**Figure 4C**). These data suggest that the methylation of the *35S* promoter under high levels of antibiotic stress is a significant factor contributing to the reduction in the expression of foreign proteins. This implied that the *35S* promoter was methylated and reduced activity, but the *LpSUT2* promoter was not (**Figure 4A**). Hence, the *LpSUT2* promoter is more advantageous for expressing foreign proteins in plants.

## 4 Discussion

Transgenic technology can rapidly improve crop yield, seed nutrient content, industrial protein production, therapeutic production, disease resistance, and abiotic stress tolerance (Somssich, 2019). Particularly, a suitable and strong promoter is decisive in initiating the expression of exogenous genes in the successful application of transgenic technology. The *35S* promoter is the most frequently used to drive transgene expression in plants (Somssich, 2019). Alternatively, the *35S* promoter also inhibits the over-expression of foreign proteins (Lang et al., 2015). Accordingly, we screened several endogenous promoters in duckweed. The results showed that the *LpSUT2* promoter had strong initiation ability and practicality, like the *35S* promoter (**Figure 1**). Increasing antibiotic stress is an effective way to improve the screening efficiency of transgenic plants. However, when using the *35S* promoter to initiate exogenous protein expression in our experiments, we found that increasing antibiotic stress reduced exogenous protein expression (**Figure 3C**). This impedes rapidly screening positive transgenic plants. On the contrary, we verified that the *LpSUT2* promoter did not reduce when increasing antibiotic stress (**Figure 3**). In our work, we developed an endogenous promoter *LpSUT2* to facilitate the rapid availability of positive plants.

**Figure 5:**
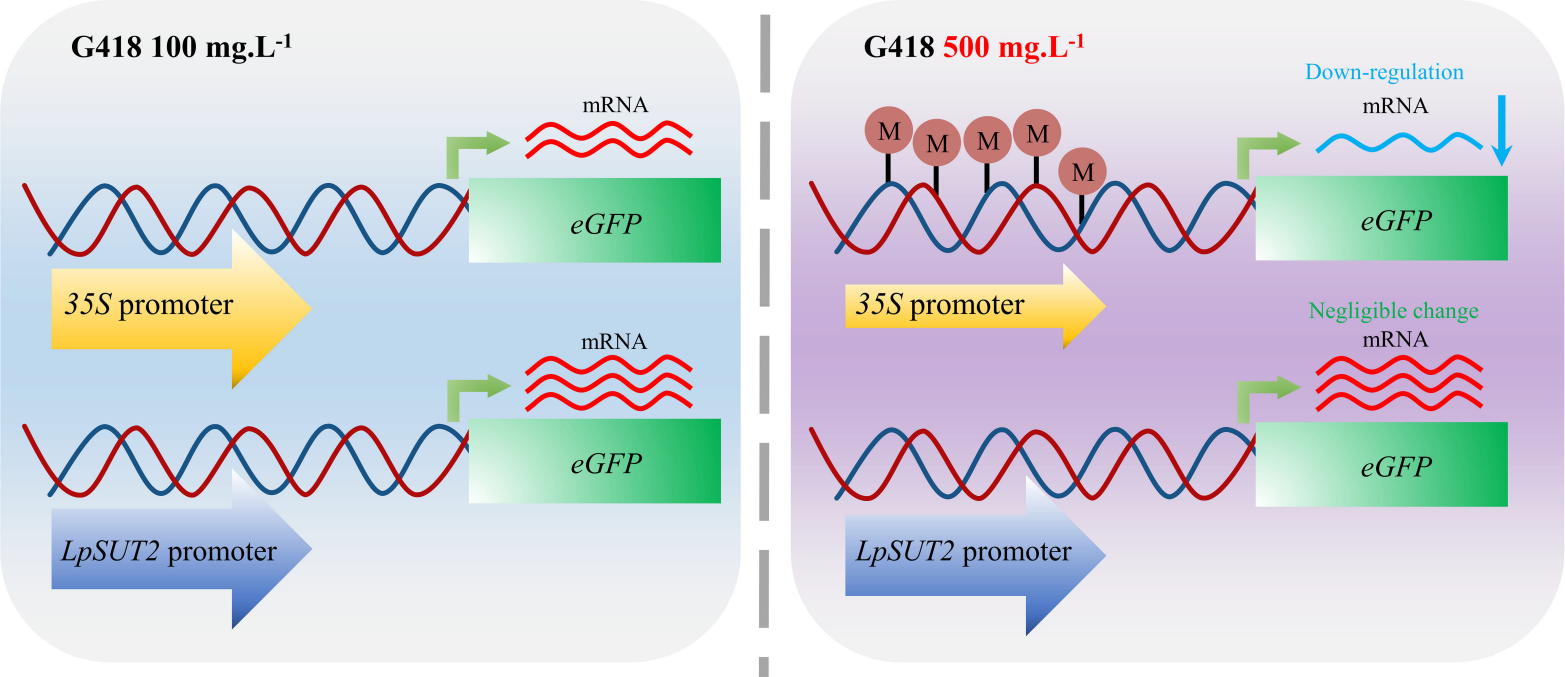
Schematic graphs of the *35S* promoter and the *LpSUT2* promoter regulating transcription under antibiotic stress. The *35S* promoter is methylated and thus down-regulates gene expression when the concentration of G418 changes from 100 mg.L^-1^ to 500 mg.L^-1^. In contrast, the *LpSUT2* promoter is not methylated and maintains high initiation activity under antibiotic stress. M, methylation.

Moreover, further experiments have been conducted to address the above interesting issue. We found that methylation was responsible for the different activities of the *35S* promoter and the *LpSUT2* promoter. The *35S* promoter was methylated after increased antibiotic stress, thereby reducing the activity of gene expression. In contrast, the *LpSUT2* promoter was not methylated and remained active under antibiotic stress (**Figure 5**). Exogenous promoters such as *35S*, often experience the silencing effects of methylation in applications (Elmayan and Vaucheret, 1996; Shimada et al., 2017). This is consistent with the decreasing activity of the *35S* promoter in increasing antibiotic stress. However, the *LpSUT2* promoter exhibits stably high activity under antibiotic stress. We also found that the *LpSUT2* promoter has more *cis*-acting elements that function like enhancers compared to the *35S* promoter, meaning it has higher activity than the *35S* promoter (**Supplementary Table 2**). In addition, compared to commonly used strong promoters (*Actin1*, *Ubi1*, and *SAG12*), the *LpSUT2* promoter also has a certain advantage in terms of the number of *cis*-acting elements. For these reasons, the *LpSUT2* promoter could be an alternative strong constitutive promoter and a very useful tool for transgenic plants.

Additionally, there may be clear distinctions between dicot and monocot in terms of the expression pattern and transcriptional activity of promoters. The *35S* promoter is a frequently utilized constitutive promoter conferring the strong expression of transgenes in dicots, while in monocots the maize ubiquitin promoter typically drives gene expression more efficiently. However, the *Ubi1* promoter is inactive in some tissues in transgenic plants, such as rice mature tissues (Cornejo et al., 1993). There are also a few promoters that displayed high promoter activity in both monocots and dicots (Jiang et al., 2018; Kummari et al., 2020), but they are not enough for the rapidly developing plant genetic engineering. In our study, we observed that the *LpSUT2* promoter from duckweed demonstrated a robust initiation ability comparable to that of the *35S* promoter during transient transformation in *Nicotiana tabacum* (dicot) (**Figure 1**). Furthermore, in the stable transformation of duckweed (monocot), the gene expression level initiated by the *LpSUT2* promoter surpassed that of the *35S* promoter (**Figure 3**). This may compensate for the limitation of transgenic promoters that are not applicable in both monocots and dicots. Together, few promoters are highly active in both monocots and dicots at present. In our work, we screened an endogenous strong promoter in duckweed and explored its underlying molecular mechanism by which it maintains high activity. The *LpSUT2* promoter has a higher potential for application in monocots and dicots compared with the *35S* promoter. Nevertheless, further experiments are required to prove whether the *LpSUT2* promoter can consistently maintain high activity in more monocots and dicots.

## 5 Conclusions

In summary, our research has discovered a novel endogenous strong promoter in duckweed and uncovered its underlying molecular mechanism. Notably, the *LpSUT2* promoter is not methylated and maintains superior activity levels compared to the commonly used *35S* promoter under antibiotic stress in monocot duckweed. Specifically, in duckweed, the expression of *eGFP* driven by the *LpSUT2* promoter was 1.76 times higher than that driven by the *35S* promoter at 100 mg.L^-1^ G418, and 6.18 times higher at 500 mg.L^-1^ G418. The *LpSUT2* promoter also exhibits a strong initiation ability similar to that of the *35S* promoter in transient transformation in dicot *Nicotiana tabacum*. This work increases the diversity of promoters and facilitates the development of plant biotechnology.

## Data availability statement

The original contributions presented in the study are included in the article/**Supplementary Material**. Further inquiries can be directed to the corresponding authors.

## Author contributions

CW: Writing – review & editing, Writing – original draft, Visualization, Validation, Software, Methodology, Investigation, Formal analysis, Data curation, Conceptualization. ZH: Writing – review & editing, Writing – original draft, Visualization, Validation, Software, Methodology, Investigation, Formal analysis, Data curation. SW: Writing – review & editing, Writing – original draft, Validation, Methodology, Formal analysis, Conceptualization. XT: Writing – review & editing, Writing – original draft, Methodology, Formal analysis. YJ: Writing – review & editing, Writing – original draft, Supervision, Project administration. ZY: Writing – review & editing, Writing – original draft, Supervision, Project administration, Funding acquisition. KH: Writing – review & editing, Writing – original draft, Methodology, Formal analysis, Conceptualization. LZ: Writing – review & editing, Writing – original draft. ZC: Writing – review & editing, Writing – original draft. YF: Writing – review & editing, Writing – original draft, Supervision, Project administration, Conceptualization. SC: Writing – review & editing, Writing – original draft, Project administration, Funding acquisition, Conceptualization. PL: Writing – review & editing, Writing – original draft, Visualization, Software, Methodology, Investigation, Formal analysis, Data curation, Conceptualization. HZ: Writing – review & editing, Writing – original draft, Supervision, Resources, Project administration, Investigation, Data curation, Conceptualization.
