# Supplementary material for: An endogenous promoter LpSUT2 discovered in duckweed: a promising transgenic tool for plants
Source: Front Plant Sci. 2024 Apr 3;15:1368284. doi: 10.3389/fpls.2024.1368284 (PMC11025394; doi:10.3389/fpls.2024.1368284)
Supplement: Supplementary file 1 [file DataSheet_1.zip › Supplementary information.pdf]

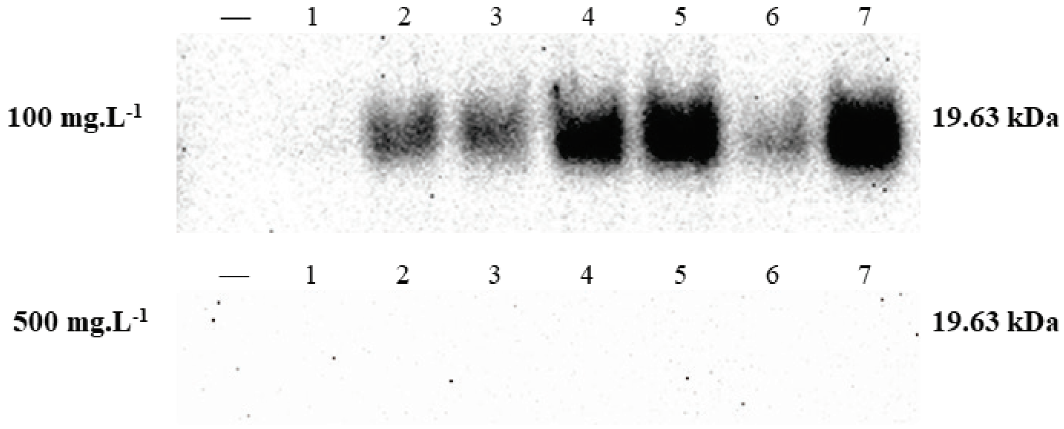

Supplementary Figure. 1 Western blot result of 35S: ChIL-2:His protein under different concentrations of G418. The 35S: ChIL-2:His protein was detected by Western blotting in transgenic duckweed under 100 mg.L^{-1} and 500 mg.L^{-1} G418. Abbreviations: -, negative control.

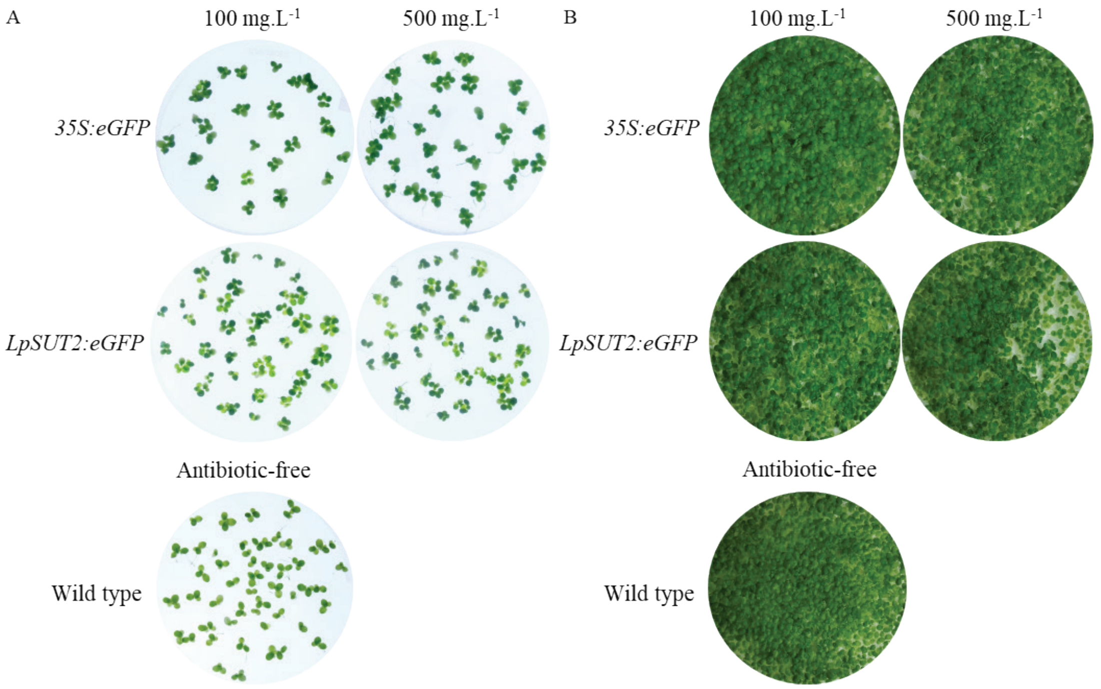

Supplementary Figure. 2 Phenotype of transgenic duckweed under different conditions. Transgenic duckweed and wild type grown in the solid agar plates (**A**) and the liquid medium (**B**) at 100 or 500 mg.L^{-1} G418, as well as antibiotic-free.

Supplementary Table 1 Primers.

Primers for Promoters	Forward (5'-3')	Reverse (5'-3')
LpSUT2	GATTCATTAATGCAGCTGAGCTCCCTCTCCTTCTTCTCCT	GCCAAGCTTATGGCACTGCAGCATCTCCAATTCAGCCTCC
LpA6804	TCATTAATGCAGCTGAACACGTCGCAACTGGCGAACACTC	GCCAAGCTTATGGCACTGCAGCATTGACGGATAGAGACCT

LpR1090	TCATTAATGCAGCTGCATGGCAGGTCGCCCTTTCATATAC	GCCAAGCTTATGGCACTGCAGCATTGGCGACTCTTCTTCT
LpR1091	TCATTAATGCAGCTGCTAGCCAGATAAACCTGCCGTCATG	GCCAAGCTTATGGCACTGCAGCATTGTTTGCCTGGGCCGG
LpU4817	TCATTAATGCAGCTGCATTCATTGGGAACACAGTTATCTC	GCCAAGCTTATGGCACTGCAGCATTGGTAACGTAGATCAA
LpU9400	TCATTAATGCAGCTGTGTTCTTAATCTCGTGGCGAATGTA	GCCAAGCTTATGGCACTGCAGCATTCTGTTAGAGAAAAAA
Primers for qRT-PCR	Forward (5'-3')	Reverse (5'-3')
eGFP	TGCAGTGCTTCAGCCGCTAC	CTTGTAGTTGCCGTCGTCCTTGA
18S	GGCGAAGGTGCGTTCAGAAGA	AGGCTGCGTCACACTACGAATT
Primers for methylation-specific PCR	Forward (5'-3')	Reverse (5'-3')
35S (M)	AAGGGTAATATTCGGAAATTTTTTC	AAATCCATCTTTAAAACCACTATCG
35S (U)	AGGGTAATATTTGGAAATTTTTTTG	CCATCTTTAAAACCACTATCAAC
LpSUT2 (M)	TTTGTAGTTTTTTTATTTTCGTCGG	ATCTCTCTAAAATCTTCTTCTCGCA
LpSUT2 (U)	TTTGTAGTTTTTTTATTTTTGTTGG	ATCTCTCTAAAATCTTCTTCTCACA

The sequence of *LpSUT2* promoter:

AGCTCCCTCTCCTTCTTCTCCTTCTCCTCAAGCCGGATGGCATTTTGTCTGGAGGAATCTCATGGAGTT
AGAGACCTGAATGCATTTCATGCTGAACATTCGGCAGTTTATATTTTATTATTAAATATACTTTGGCAGTT
TCAGAACAATAAGGCAACGTAAATTACAATGGCTTCGTACTTGTTTGACATCTATGCAGGAATATTAAA
CATGCAATGAATTTACATGAATCTGCTCTTTAGATCAGCATGTTCTCTTTCTCTATTTCTCTCTCTTGTGT
ATGAAAGAGAAGCAAAATGGAAGAAGATAATCATAATCTTTACAAGGAGAATGGAGAAGCACGTAGA
AAGGAGAGCTTTCTTGCACTAGTTCGAAAACCATGGATGGGGCAGAGTTGGGAACCGATTGATTTCCT
CTAGTTCGAAAACCAGAGATATTTTAGCCGGCACACGTTTTGAGAACGAAAAGAATCACCCGTGTCGG
CGAATTTCAACCTCTTGGTGGGAAAATGAGTGAATCGCGATAGAGAAGAAATCAGTAATTTTATAAGTA
AACATACAAAATGATGCACAGAAGGAAATCATGGAGGCAATGTCATCTGGCACAGAATGATATTGAAA
TAGGAAGAAGAATGAAGAAAATATGAATAGAGATTCAACAAATCTATGCATGTTTAGGAAAAAAAAAA
AAGAAATTTGAAGCAATTCAAAATTTTAGAAATCAGATCTAGCGAGGCACGGAATCAAAAAACAATGT
TAAGTACCAACCGGCGCCACTCTCTGAGGATAAATCCTTCTTCGGACTGCATCTCAATTGGAGGTGGA
AGGATTGGACCATCTGACACGAAAACCCCTCCATCATCCGCCTCCTCATACGGCTTTCCATTAGTCTCC
GGCATCATAAATGGCGAGGGAACGGAGGAAAAGTCCGGATCCGGCTCAGATCGCAGCCCAAAGTTCT
CAGGCGAAGGCGGGATGCTGTCCCCAGAAACTACTGGAATGGGGATATCTTCGTCCAGCCCGAACCCG
CCGCCGCCGTGGCCATGATCGGACGGAAAACCCAGAGGGGGCTCCTCGGAGGGAATTCCGCCGACAA
AATCCTTACCATCTGCGTCACTGCCGAAGCCGTTGTAGGATTCGAATCGCTGGGACGGAAGGCGCGGA
TCATAACCGAGGTAGCCATCATCGAAGGGCCGCGCAGATCCCCTTGCAGCTCCCTCATCCCCGTCGGT
GGTGAAGGCGTCGAATGAAGAAGACATCTCGCCTGCTCCCGATCTCTCCCACTTCCTCCCCTCTCCGAT

CAGTCTCCGCTCTCGCCGCCCCGCGTCTCCGATCTGGAAGCAGCTTTATCACGGTGGCTGCGAGAAGA
AGACCTCAGAGAGACATTTCGTTACGTTTACCGTCCGATCGGTGGACCAAGCCGAGATCATCATGCAA
CATGAAATTGAAATAATTTAGTAATTACCCATTACTCGAATTAATTATTTTAAAATTGCTGAGTGTGCTGT
TCTACGGATCGGCTCCCCACCTGGCGAGTCCCGATTCCAGTCCAGAATCGGTCCGAGTCGAGCTCTCT
AACTCGGAGGAAGATCGAGTGTTCATGAACCCCGCCACCGCCGTAGACACGGGAAAGAATTCCGCTC
CGTTCGGTGGATCCTGATCTCGATCGCTGAGGAAATCGGAACCGTGCCGGCCGGAGGGAGATGCTTTC
ACGGCCGCTGAAGTGCATATCCATCGCCTTCCATAGCTCTGAAACTCTCGGTTTCGCAGCCGAGAACA
GTGGTCTAATTCAGAGCCCCGCCTTCAGCGTAGCGCCGCTGCGGATCGACTGTGTTGATCTAAGTGGA
AAATCTAGAAACCCTAACGAGGTATTTGCGAATCGGAGAGCTTCCTGATGCTGTGTTCTTCACGTGCAT
CCCTGTTTGTACGCTCTTTTGTGCAGTAATGGACGGGTTCTCGAGCCGCGTGCCGTACCGGCATCTGAA
GGAGGCTGAATTGGAG

Cis-acting elements of the *35S* and the *LpSUT2* promoter

The *cis*-acting element, a DNA sequence in the promoter region that binds to transcription factors to regulate initiation and transcription. We analyzed the *cis*-acting elements of the *35S*, *LpSUT2*, *Actin1*, *Ubi1*, and *SAG12* promoters using the PlantCARE database (http://bioinformatics.psb.ugent.be/webtools/plantcare/html/). Next, we compared and found that the numbers of CAAT-box and G-box *cis*-acting elements in the *LpSUT2* promoter region (20 and 3 respectively) were more than those in the *35S* promoter region (11 and 2 respectively), compared with other promoters such as *Actin1* promoter region (21 and 3, respectively), *Ubi1* promoter region (11 and 3, respectively), and *SAG12* promoter region (38 and 0, respectively), it also possesses distinct advantages and characteristics (Supplementary Table 2). These *cis*-acting elements can enhance the activities of promoters when combined with transcription factors (Almeyda et al., 2015; Baum et al., 1999), and the activities of promoters are related to the copy number of the *cis*-acting elements (Qian et al., 2007). Therefore, our results showed that the *35S, LpSUT2, Actin1, Ubi1, and SAG12* promoters had *cis*-acting elements that could enhance the activity of the promoter. These elements in the *LpSUT2* promoter were more than those in the *35S* promoter, and the *LpSUT2* promoter shares similarities with other plant endogenous strong promoters in terms of its characteristics.

References

Almeyda, C. V., Raikhy, G., and Pappu, H. R. (2015). Characterization and comparative analysis of promoters from three plant pararetroviruses associated with *Dahlia* (*Dahlia variabilis*). *Virus Genes* 51, 96–104. doi: 10.1007/s11262-015-1196-7.

Baum, K., Wienand, U., and Meier, I. (1999). Reduction of G-box binding factor DNA binding activity, but not G-box binding factor abundance, causes the downregulation of RBCS2 expression during early tomato fruit development. *FEBS Lett.* 454, 95–99. doi: 10.1016/S0014-5793(99)00784-X.

Qian, W., Tan, G., Liu, H., He, S., Gao, Y., and An, C. (2007). Identification of a bHLH-type G-box binding factor and its regulation activity with G-box and Box I elements of the *PsCHS1* promoter. *Plant Cell Rep.* 26, 85–93. doi: 10.1007/s00299-006-0202-x.
