# Supplementary figures and images for: An endogenous promoter LpSUT2 discovered in duckweed: a promising transgenic tool for plants
Source: Front Plant Sci. 2024 Apr 3;15:1368284. doi: 10.3389/fpls.2024.1368284 (PMC11025394; doi:10.3389/fpls.2024.1368284)

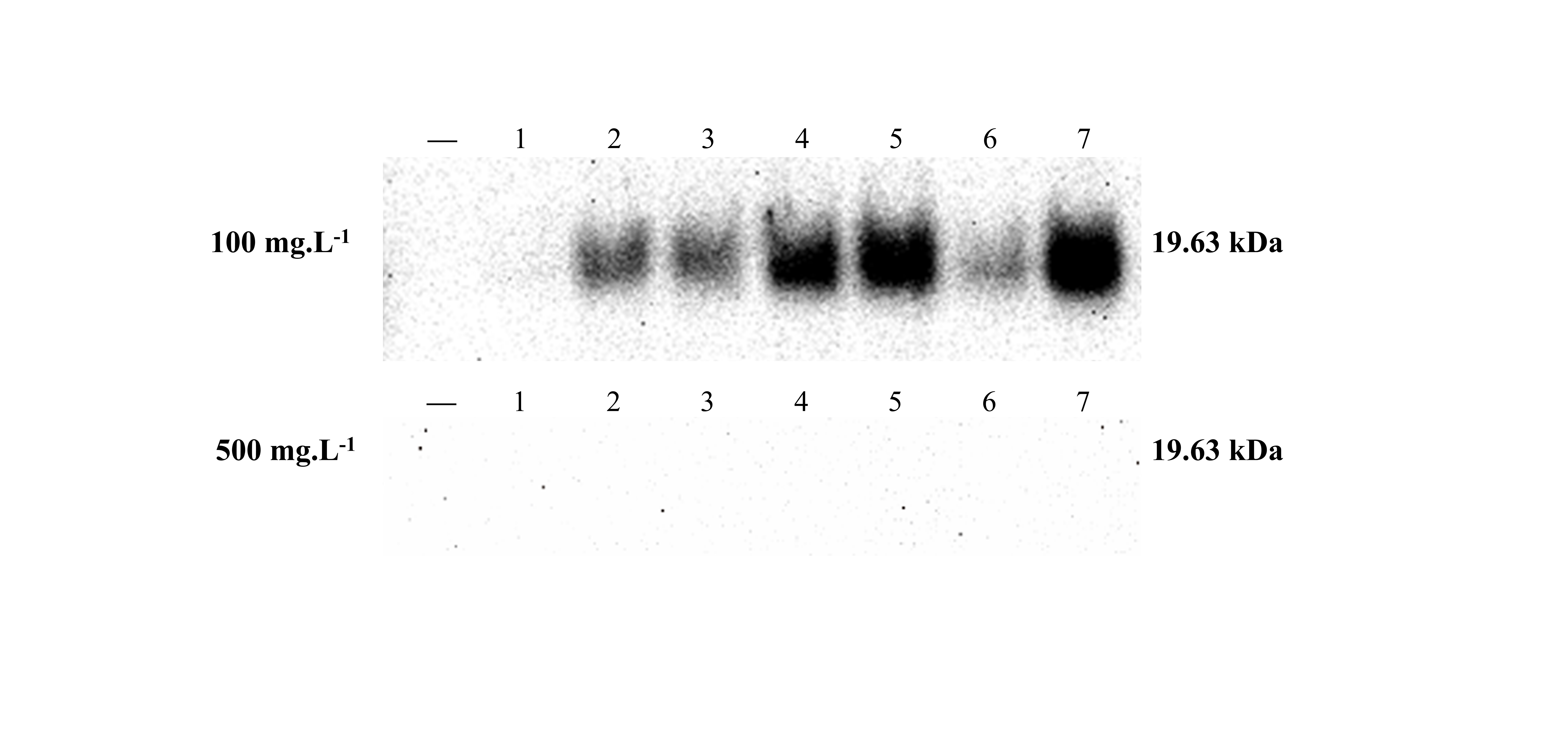

Supplement: Supplementary file 1 [file DataSheet_1.zip › Supplementary Figure1.tif]

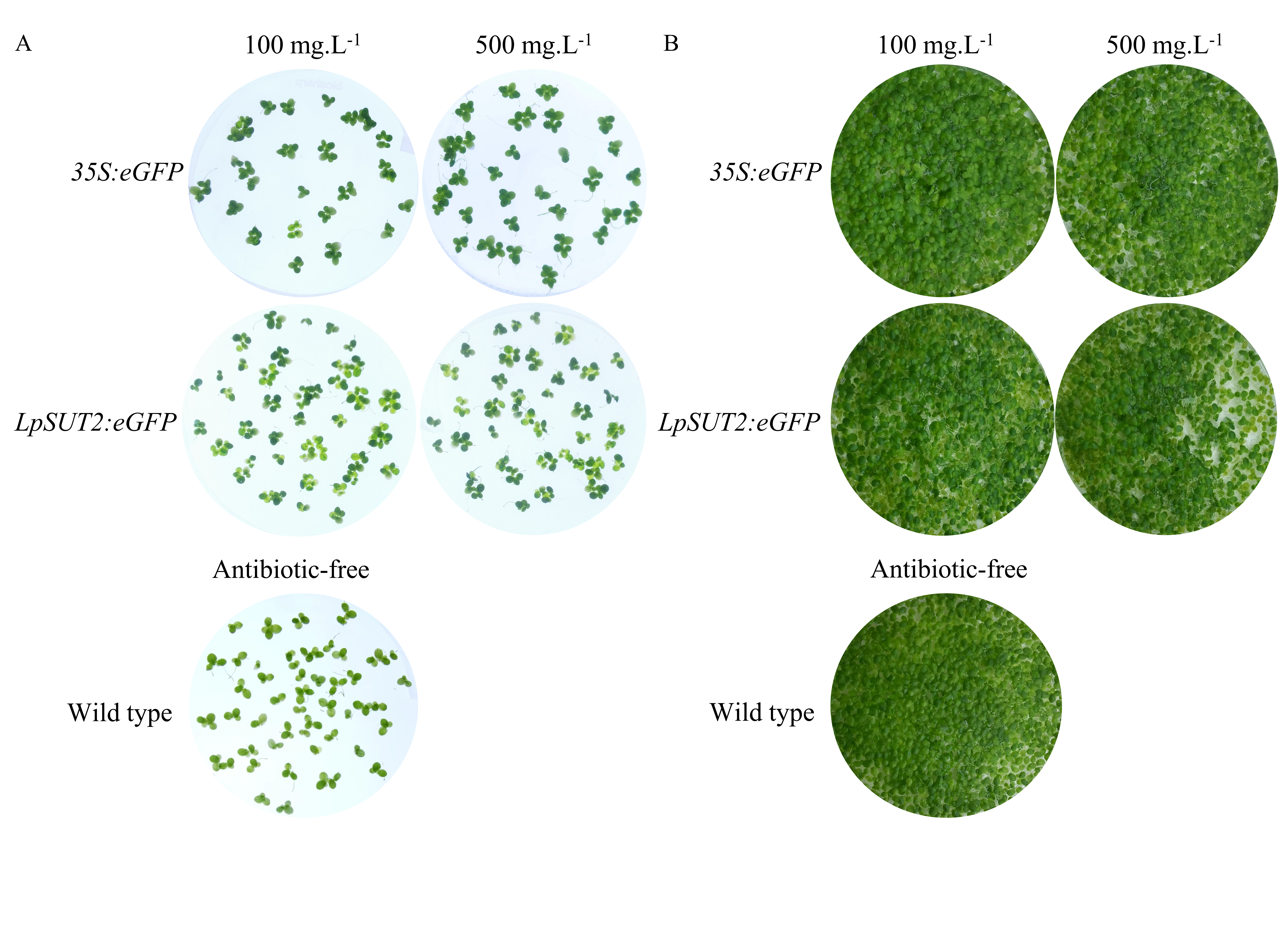

Supplement: Supplementary file 1 [file DataSheet_1.zip › Supplementary Figure2.tif]
